# Noninsulin‐based antihyperglycemic medications in patients with diabetes and COVID‐19: A systematic review and meta‐analysis

**DOI:** 10.1111/1753-0407.13359

**Published:** 2023-01-23

**Authors:** Mahmoud Nassar, Hazem Abosheaishaa, Awadhesh Kumar Singh, Anoop Misra, Zachary Bloomgarden

**Affiliations:** ^1^ Department of Medicine Icahn School of Medicine at Mount Sinai/NYC Health+Hospitals/Queens New York City New York USA; ^2^ Department of Diabetes & Endocrinology GD Hospital & Diabetes Institute Kolkata India; ^3^ Chairman, Fortis‐C‐DOC Centre of Excellence for Diabetes, Metabolic Diseases and Endocrinology, Diabetes Foundation (India), and National Diabetes Obesity and Cholesterol Foundation (NDOC) New Delhi India; ^4^ Department of Medicine, Division of Endocrinology, Diabetes and Bone Disease Icahn School of Medicine at Mount Sinai New York New York USA

**Keywords:** COVID‐19, diabetes, DPP‐4 inhibitors, GLP‐1RA, oral antihyperglycemic, SGLT‐2 inhibitors, sulfonylureas, thiazolidinediones, 口服降糖药物, 新型冠状病毒肺炎, 糖尿病, GLP‐1RA, SGLT‐2抑制剂, 磺脲类, 噻唑烷二酮类, DPP‐4抑制剂

## Abstract

**Background:**

Patients with diabetes are more likely to suffer COVID‐19 complications. Using noninsulin antihyperglycemic medications (AGMs) during COVID‐19 infection has proved challenging. In this study, we evaluate different noninsulin AGMs in patients with COVID‐19.

**Methods:**

We searched Medline, Embase, Web of Science, and Cochrane on 24 January 2022. We used the following keywords (COVID‐19) AND (diabetes mellitus) AND (antihyperglycemic agent). The inclusion criteria were studies reporting one or more of the outcomes. We excluded non‐English articles, case reports, and literature reviews. Study outcomes were mortality, hospitalization, and intensive care unit (ICU) admission.

**Results:**

The use of metformin rather than other glucose‐lowering medications was associated with statistically significant lower mortality (risk ratio [RR]: 0.60, 95% confidence interval [CI]: 0.47, 0.77, *p* < .001). Dipeptidyl peptidase‐4 inhibitor (DPP‐4i) use was associated with statistically significantly higher hospitalization risk (RR: 1.44, 95% CI: 1.23, 1.68, *p* < .001) and higher risk of ICU admissions and/or mechanical ventilation vs nonusers (RR: 1.24, 95% CI: 1.04, 1.48, *p* < .02). There was a statistically significant decrease in hospitalization for SGLT‐2i users vs nonusers (RR: 0.89, 95% CI: 0.84–0.95, *p* < .001). Glucagon‐like peptide‐1 receptor agonist (GLP‐1RA) use was associated with a statistically significant decrease in mortality (RR: 0.56, 95% CI: 0.42, 073, *p* < 0.001), ICU admission, and/or mechanical ventilation (RR: 0.79, 95% CI: 0.69–0.89, *p* < .001), and hospitalization (RR: 0.73, 95% CI: 0.54, 0.98, *p* = .04).

**Conclusions:**

AGM use was not associated with increased mortality. However, metformin and GLP‐1RA use reduced mortality risk statistically significantly. DPP‐4i use was associated with a statistically significant increase in the risk of hospitalization and admission to the ICU.

## Graphical abstract

**Highlights**

- Metformin was associated with statistically significantly lower overall mortality for inpatients and outpatients.
- Dipeptidyl peptidase‐4 inhibitor use was associated with statistically significant higher hospitalization risk and higher risk of intensive care unit (ICU) admissions and/or mechanical ventilation.
- There was a statistically significant decrease in hospitalization for sodium glucose transporter 2 inhibitor) users vs. nonusers.
- Glucagon‐like peptide‐1 receptor agonist use was associated with a statistically significant decrease in mortality, ICU admission and/or mechanical ventilation, and hospitalization.

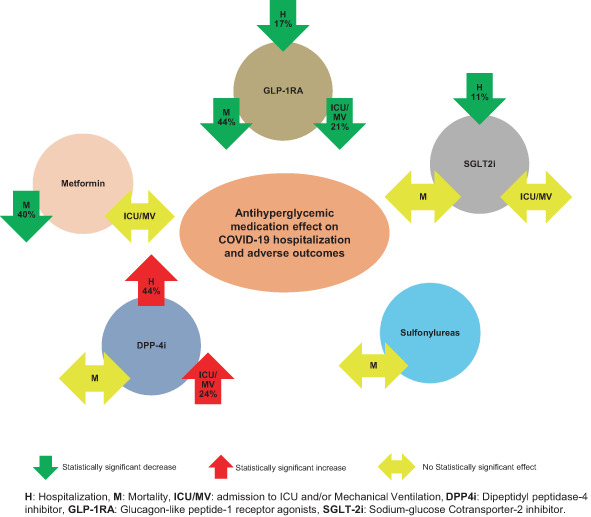

## 摘要

**背景:**糖尿病患者更易罹患COVID‐19并发症。在COVID‐19感染期间使用非胰岛素降糖药物(AGMs)具有挑战性。在本研究中, 我们评估了COVID‐19患者的不同非胰岛素降糖药物。

**方法:**检索Medline、Embase、Web of Science和Cochrane数据库, 检索时间截至2022年1月24日。我们使用了以下关键词:COVID‐19、糖尿病和降糖药。纳入标准为报告一项或多项结局的研究, 并排除非英文文献、病例报告和文献综述。研究结局包括死亡率、住院和入住ICU。

**结果:**与其他降糖药物相比, 使用二甲双胍与显著较低的死亡率相关(风险比[RR] 0.60,95% CI 0.47,0.77,p<0.001)。与非使用者相比, 使用DPP‐4i与较高的住院风险(RR 1.44, 95% CI 1.23, 1.68, p<0.001)和较高的ICU入住和/或机械通气风险(RR 1.24, 95% CI 1.04, 1.48, p<0.02)相关。SGLT‐2i用药者的住院率显著低于非用药者(RR 0.89, 95% 0.84 ‐0.95, p <0.001)。胰高血糖素样肽‐1受体激动剂(GLP‐1RA)的使用与死亡率(RR 0.56, 95%CI, 0.42, 073, p<0.001)、入住ICU和/或机械通气(RR 0.79, 95%CI, 0.69‐ 0.89, p<0.001)和住院(RR 0.73, 95%CI 0.54, 0.98, p=0.04)显著降低相关。

**结论:**AGMs的使用与死亡率增加无关。二甲双胍和GLP‐1RA的使用显著降低了死亡率风险。DPP‐4i的使用与住院和入住ICU的风险显著增加相关。

## 1 INTRODUCTION

Coronavirus disease 2019 (COVID‐19), caused by SARS‐CoV‐2, is a worldwide pandemic that has caused more than 586 million cases and 643 million deaths (as of August 2022).^1^ Clinically, COVID‐19 can manifest as a wide range of symptoms, from asymptomatic to severe. Diabetes is associated with an increased risk of developing more severe COVID‐19 and a greater risk of hospitalization, intensive care unit (ICU) admission, and mortality.^2^, ^3^ Furthermore, recommended treatment with corticosteroids increases the risk of uncontrolled glycemia.^2^ The decision to continue using antihyperglycemic medications (AGMs) in patients with diabetes is challenging. This meta‐analysis aims to study the impact of different groups of AGMs on patients with COVID‐19. A comparison was made between those receiving the AGMs and those not receiving them.

## 2 METHODS

We searched Medline/PubMed, Embase, Web of Science, and Cochrane on January 24, 2022. We used the following keywords (COVID‐19 OR Coronavirus disease 2019 [MESH]) AND (Diabetes mellitus) AND (Oral antihyperglycemic agent OR sulfonylurea derivative OR Dipeptidyl peptidase‐4 inhibitor Or Sodium‐glucose cotransporter 2 Or Empagliflozin Or dapagliflozin OR Canagliflozin OR Metformin OR Glucagon‐like peptide‐1 receptor agonist pioglitazone OR hypoglycemic agents).

We included in this analysis studies that included patients with type 2 diabetes (T2D) who were infected with COVID‐19. The present meta‐analysis examines the effect of different AGMs on mortality, hospitalization, and admission to the ICU and/or mechanical ventilation. Each category of AGM is compared with the other groups. We included studies that reported one or more of the following outcomes: mortality, hospitalization, ICU admission, or mechanical ventilation. We excluded systematic reviews, non‐English articles, case reports, case series, literature reviews, posters, abstracts, pediatric and pregnancy cases, and patients receiving insulin for outpatient treatment. The term “mortality” was defined as death occurring within 30 days of confirmed infection with COVID‐19. The identified articles were uploaded to the Covidence website for removal of duplicate files and screening. Coauthors independently screened, reviewed, and extracted the data from the full text. We extracted the data into a spreadsheet in Microsoft Excel. An evaluation of the risk of bias was carried out by two independent coauthors using the GRADE assessment for randomized controlled trial (RCT) (Figure S25) and Newcastle‐Ottawa quality assessment for retrospective cohort studies (Table S1). The study did not involve human or animal subjects.

## 3 RESULTS

**FIGURE 1:**
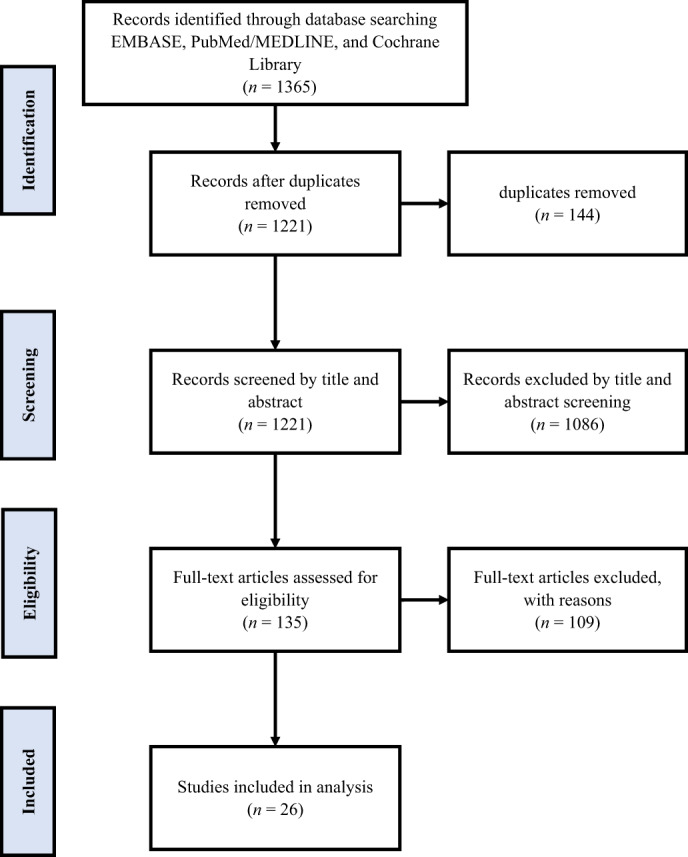
Preferred Reporting Items for Systematic Reviews and Meta‐Analyses (PRISMA) flow diagram of the literature search.

A total of 1365 articles were found after removing 144 duplicates. A total of 1221 articles were screened based on the title and abstract. A total of 1086 articles were excluded at this stage. We screened 135 articles based on their full text, and 108 articles were excluded. This study included 26 articles, shown in Preferred Reporting Items for Systematic Reviews and Meta‐Analyses (PRISMA) Figure 1 and Table 1.

**TABLE 1:** Characteristics of included studies

Study	Drug	Country	Duration	Study type	No. of patients
Cariou et al,^4^	Metformin Sulfonylurea/glinides DPP‐4i, GLP1‐RA	French	10–31 March 2020.	Retrospective 53 French centers	1317
Chen et al,^5^	DPP‐4i, metformin, alpha glucosidase	China	November 2020 to March 2020.	Retrospective single center	904
Do et al,^6^	Metformin	Korea	February 2020 to May 2020.	Retrospective	1865
Elibol et al,^7^	Oral antihyperglycemic	Turkey	March 2020 to September 2020.	Cross‐sectional	432
Emral et al,^8^	DPP‐4i	Turkish	March 2020 to May 2020.	Retrospective, multicenter, national electronic data	33 478
Fadini et al,^9^	DPP‐4i	Italy	February 2022 to April 2020.	Retrospective single center	403
Ghany et al,^10^	Metformin	United States	January 2020 to August 2020.	Retrospective multicenter	1139
Israelsen et al,^11^	SGLT‐2i	Denmark	February 2020 to November 2020.	Retrospective	1970
Jiang et al,^12^	Metformin	China	December 2019 to March 2020.	Retrospective	328
Kahkoska et al,^13^	GLP‐1RA, SGLT‐2i	United States	January 2018 to February 2021.	Multicenter, longitudinal	12 446
Kosiborod et al,^14^	Dapagliflozin	Argentina, Brazil, Canada, India, Mexico, the United Kingdom, and the United States.	April 2020 to January 2021.	Randomized, double‐blind, placebo‐controlled trial	1250
Lalau et al,^15^	Metformin	French	March 2020 to April 2020.	Retrospective multicenter study, 68 French centers	2449
Luk et al,^16^	Metformin	Hong Kong	January 2020 to February 2021.	Retrospective	1220
Luo et al,^17^	Metformin	China	January 2020 to March 2020.	Retrospective observational	283
Meijer et al,^18^	DPP‐4i	Netherlands	March 2020 to October 2020.	Prospective cohort study, Covid Predict Clinical Course Cohort, multicenter	565
Mirani et al,^19^	DPP‐4i	Italy	February 2020 to April 2020.	Retrospective single center	90
Noh et al,^20^	DPP‐4i	Korea	January 2017 to May 2020.	Cohort study	586
Nyland et al,^21^	GLP‐1RA	Multinational	January 2020 to September 2020.	Multinational retrospective cohort study/TriNetX COVID‐19 Research Network of 56 large healthcare organizations	29 516
Ong et al,^22^	Metformin	Philippines	March 2020 to September 2020.	Retrospective	355
Perez‐Belmonte et al,^23^	DPP‐4i, metformin	Spain	March 2020, to July 2020.	Nationwide cohort study	2666
Ramos‐Rincón et al,^24^	DPP‐4i, metformin, SGLT‐2i, GLP‐1RA	Spain	March 2020 to May 2020.	Nationwide observational study	790
Silverii et al,^25^	DPP‐4i, metformin, sulphonylurea, SGLT‐2i, GLP‐1RA	Sicily	March 2020 to May 2020.	Retrospective observational study	159
Solerte et al,^26^	DPP‐4i	Italy	March 2020 to April 2020.	In a multicenter, case–control, retrospective, observational study	338
Sourij et al,^27^	DPP‐4i, metformin, sulphinylurea, SGLT‐2i, GLP‐1RA	Austria	April 2020 to June 2020.	combined prospective and retrospective, multi‐center	247
Wargny et al,^28^	DPP‐4i, metformin, sulphonylurea, GLP‐1RA	France	March 2020 to April 2020.	Prospective nationwide multicenter study	2796
Wong et al,^29^	DPP‐4i	Hong Kong	January 2020 to January 2021.	Retrospective	1214

Abbreviations: DPP‐4i, dipeptidyl peptidase‐4 inhibitor; GLP‐1RA, glucagon‐like peptide‐1 receptor agonists; SGLT‐2i, sodium‐glucose cotransporter‐2 inhibitor.

### 3.1 Metformin

#### 3.1.1 Mortality

This analysis included 15 studies involving 6185 patients who used metformin as part of their treatment.^5^, ^6^, ^7^, ^10^, ^12^, ^15^, ^16^, ^17^, ^19^, ^22^, ^23^, ^24^, ^25^, ^27^, ^28^ The pooled analysis showed that metformin was associated with statistically significant lower mortality than other AGMs (risk ratio [RR]: 0.60, 95% confidence interval [CI]: 0.47, 0.77, *p* < .001) with substantial heterogeneity *I* ^2^ = 83% (Figure S1 and S2).

#### 3.1.2 ICU admission and/or mechanical ventilation

Four studies assessed the effects of metformin on ICU admission and/or mechanical ventilation,^6^, ^15^, ^16^, ^23^ finding no difference between metformin users vs nonusers (RR: 0.96, 95% CI: 0.63, 1.44, *p* ≤ .83), although with considerable heterogeneity *I* ^2^ = 89% (Figure S3 and S4).

### 3.2 Dipeptidyl peptidase‐4 inhibitor mortality and hospitalization statistics

#### 3.2.1 Mortality

We included 18 studies in the analysis.^4^, ^5^, ^7^, ^8^, ^9^, ^11^, ^13^, ^18^, ^19^, ^20^, ^21^, ^23^, ^24^, ^25^, ^26^, ^27^, ^28^, ^29^ No difference in mortality was observed with Dipeptidyl peptidase‐4 inhibitor (DPP‐4i) users vs nonusers (RR: 1.10, 95% CI: 0.89, 1.37, *p* < .37), although this outcome exhibited substantial heterogeneity (*I* ^2^ = 89%, *p* < .01) (Figures S5 and S6).

#### 3.2.2 Hospitalization

Four studies were included in the analysis of hospitalization.^8^, ^11^, ^13^, ^21^ The pooled data showed that DPP‐4i was associated with a statistically significantly higher risk of hospitalization than those not using DPP‐4i (RR: 1.44, 95% CI: 1.23, 1.68, *p* < .001) with considerable heterogeneity (*I* ^2^ = 95%, *p* < .001) (Figures S7 and S8).

#### 3.2.3 ICU admission and/or mechanical ventilation

Seven studies are included in the analysis of ICU admission and/or mechanical ventilation.^8^, ^11^, ^13^, ^18^, ^23^, ^26^, ^29^ The pooled finding indicated DPP‐4i was associated with a statistically significant increase in ICU admission and/or mechanical ventilation in comparison with nonuse of DPP‐4i (RR: 1.24, 95% CI: 1.04, 1.48, *p* = .02) with substantial heterogeneity (*I* ^2^ = 68%, *p* = .005) (Figure S9 and S10).

### 3.3 Sulfonylureas/meglitinides

#### 3.3.1 Mortality

Five studies^7^, ^19^, ^25^, ^27^, ^28^ studied mortality outcomes with sulfonylureas or meglitinides. No difference in mortality was observed with sulfonylureas/meglitinides (RR: 0.96, 95% CI: 0.54, 1.70, *p* ≤ .88) compared to nonusers, although with substantial heterogeneity (*I* ^2^ = 86%, *p* < .001) (Figures S11 and S12).

### 3.4 Sodium‐glucose cotransporter‐2 inhibitors (SGLT‐2i)

#### 3.4.1 Mortality

Seven studies^7^, ^11^, ^13^, ^14^, ^24^, ^25^, ^27^ included information on mortality outcomes. No difference in mortality was observed in SGLT‐2i users vs nonusers (RR: 0.82, 95% CI: 0.65, 1.04, *p* = .11) (Figures S13 and S14).

#### 3.4.2 ICU admission and/or mechanical ventilation

The pooled analysis of two studies^11^, ^13^ indicated no difference in ICU admission and/or mechanical ventilation in SGLT‐2i users (RR: 0.91, 95% CI: 0.78, 1.06, *p* = .21) vs nonusers, with no heterogeneity (*I* ^2^ = 0%, *p* = .85) (Figures S15 and S16).

#### 3.4.3 Hospitalization

Pooled data from two studies^11^, ^13^ showed that SGLT‐2i users were significantly less likely to require hospitalization (RR: 0.89, 95% CI: 0.84–0.95, *p* < .001) compared with nonusers, with no heterogeneity (*I* ^2^ = 0%, *p* = .37) (Figures S17 and S18).

### 3.5 Glucagon‐like peptide‐1 receptor agonists (GLP‐1RAs)

#### 3.5.1 Mortality

Seven studies^11^, ^13^, ^21^, ^24^, ^25^, ^27^, ^28^ pooled in this meta‐analysis showed GLP‐1RAs were associated with statistically significant lower mortality in comparison with the non‐GLP‐1RA group (RR: 0.56, 95% CI: 0.42, 073, *p* < .001), with significant heterogeneity (*I* ^2^ = 60%, *p* = .02) (Figures S19 and S20).

#### 3.5.2 Hospitalizations

Pooled analysis of three studies^11^, ^13^, ^21^ showed GLP‐1RA use was associated with a statistically significant reduction in hospitalizations (RR: 0.73, 95% CI: 0.54, 0.98, *p* = .04), with substantial heterogeneity (*I* ^2^ = 94%, *p* < .001) (Figures S21 and S22).

#### 3.5.3 ICU admission and/or mechanical ventilation

Two studies^11^, ^13^ reported ICU admission and/or mechanical ventilation. Pooled analysis showed GLP‐1RA users had a statistically significant lower likelihood of ICU admission and/or mechanical ventilation (RR: 0.79, 95% CI: 0.69–0.89, *p* < .001), with no heterogeneity (Figures S23 and S24). The results of the AGMs COVID‐19 outcome meta‐analysis are summarized in Table 2.

**TABLE 2:** Summary of the antihyperglycemic medications (AGM) COVID‐19 outcome meta‐analysis

Medication	Outcome	Events	Total	Comparator events	Comparator total	Risk ratio (random, 95% CI)
Metformin	Hospitalization	NA	NA	NA	NA	NA
	ICU/mechanical ventilation	577	2978	314	1551	0.96 [0.63,1.44]
	Mortality	3225	6185	1165	4670	0.60 [0.47,0.77]
DPP‐4i	Hospitalization	6491	12 900	16 893	39 837	1.44 [1.23, 1.68]
	ICU/mechanical ventilation	1433	10 995	3769	39 471	1.24 [1.04, 1.48]
	Mortality	1552	15 261	3764	47 095	1.10 [0.89, 1.37]
Sulfonylurea	Hospitalization	NA	NA	NA	NA	NA
	ICU/mechanical ventilation	NA	NA	NA	NA	NA
	Mortality	185	905	662	2697	0.96 [0.54, 1.70]
SGLT‐2i	Hospitalization	905	3908	2816	10 841	0.89 [0.84, 0.95]
	ICU/mechanical ventilation	235	3911	715	10 855	0.91 [0.78, 1.06]
	Mortality	171	4652	1019	13 122	0.82 [0.65, 1.04]
GLP‐1RA	Hospitalization	1826	8828	2937	10 428	0.73 [0.54, 0.98]
	ICU/echanical ventilation	399	7062	551	7704	0.79 [0.69, 0.89]
	Mortality	241	9124	1465	14 078	0.56 [0.42, 0.73]

Abbreviations: CI, Confidence interval; DPP4i, dipeptidyl dipeptidyl peptidase‐4 inhibitor; GLP‐1RA, glucagon‐like peptide‐1 receptor agonists; ICU, intensive care unit; NA, not available due to insufficient data; SGLT‐2i, sodium‐glucose cotransporter‐2 inhibitor.

## 4 DISCUSSION

We conducted a meta‐analysis based on pooled data from 26 primary studies.^4^, ^5^, ^6^, ^7^, ^8^, ^9^, ^10^, ^11^, ^12^, ^13^, ^14^, ^15^, ^16^, ^17^, ^18^, ^19^, ^20^, ^21^, ^22^, ^23^, ^24^, ^25^, ^26^, ^27^, ^28^, ^29^ A higher risk of severe COVID‐19 and mortality is associated with comorbidities such as cardiometabolic disease and diabetes.^30^, ^31^ Several AGMs have been studied, including etformin, DPP‐4i, sulfonylureas, glinide, SGLT‐2i, and GLP‐1RA. Statistically significant findings include the following: metformin and GLP‐1RA use were associated with statistically significant reductions in mortality risk, DPP‐4i use was associated with a statistically significant increase in the likelihood of hospitalization and admission to ICU, and SGLT‐2i use was associated with a statistically significant reduction in hospitalization risk. These findings have important implications for clinical practice. However, more robust studies, particularly RCTs, are needed.

In the metformin group, pooled analysis of 15 included studies revealed its use to be associated with decreased mortality compared to nonuse of metformin.^5^, ^6^, ^7^, ^10^, ^12^, ^15^, ^16^, ^17^, ^19^, ^22^, ^23^, ^24^, ^25^, ^27^, ^28^ Our results agree with the findings of Scheen,^32^ who noted that metformin has a complex mechanism of action, some of which leads to anti‐inflammatory activity that may help reduce the risk of severe COVID‐19 beyond the effects of glucose control.^10^, ^33^ Nuclear factor kappa‐light‐chain‐enhancer of activated B cells nuclear factor‐kB (NF‐κB) and mammalian target of rapamycin are two other pathways that may be affected by metformin to reduce the release of inflammatory markers.^32^ Further, metformin affects SARS‐CoV2 replication and virus entry into the cell by phosphorylating the angiotensin‐converting enzyme 2 (ACE2) receptor via adenosine monophosphate‐activated protein kinase.^34^, ^35^ Finally, metformin has a role in other viral infections, such as stimulating autophagy in neuroblastoma cells and reducing herpes simplex virus type 1 (HSV‐1) propagation.^36^ In patients with dengue infection, metformin regulates immune‐metabolic activities.^37^

A mechanism for the observed increase in the risks of both hospitalization and ICU admission and/or mechanical ventilation in patients using DPP‐4i compared to those not using DPP‐4i is not entirely apparent. There may be the proinflammatory activity of DPP‐4i, which might contribute to the hyperinflammation associated with COVID‐19,^38^ although others have suggested that DPP‐4i exhibits direct anti‐inflammatory, immunomodulatory, and antifibrotic effects.^39^, ^40^, ^41^ As a receptor, DPP‐4 may play a role in SARS‐CoV‐2 entry into the host.^42^

Although the spike protein of the SARS‐CoV‐2 virus does not interact with human membrane‐bound DPP‐4 (CD26),^43^, ^44^ patients with T2D are thought to have dysregulated DPP‐4 levels, which might have a negative vascular impact, resulting in an increased risk of COVID‐19.^45^ DPP‐4 is present in visceral fat, contributing to insulin resistance and adipocyte inflammation, so that DPP‐4i might correct some of the factors linking obesity with poor outcomes.^46^

Many types of immune cells express DPP‐4, including CD4(+) and CD8(+) T cells, B cells, NK cells, dendritic cells, and macrophages.^44^, ^47^ By activating the NF‐kB signaling pathway, DPP‐4 promotes the activation and proliferation of T cells.^48^, ^49^ There is evidence that DPP‐4's effects are triggered by interactions between antigen‐presenting cells and markers such as CD45, caveolin‐1, mannose‐6 phosphate receptors, or adenosine deaminase (ADA). Owing to its interaction with ADA, DPP‐4 facilitates immune cell migration and diapedesis.^45^ As a result of reducing cytokine storms, DPP‐4i plays a role in preventing acute respiratory distress syndrome.^48^, ^50^, ^51^ In addition, DPP‐4i has also been speculated to have antifibrotic effects because DPP‐4 is known to stimulate the production of cytokines and chemokines by fibroblasts and the proliferation of smooth muscle cells. Therefore, DPP‐4i may prevent lung fibrosis progression and reduce mechanical complications associated with COVID‐19.^44^

Based on a pooled analysis of five studies,^7^, ^19^, ^25^, ^27^, ^28^ there were no statistically significant differences in mortality between patients treated with sulphonylureas/glinides and those treated with non‐sulphonylureas. Other studies suggest that sulfonylureas used prior to admission were associated with a borderline increased risk of adverse outcomes during hospitalization.^52^

Some studies recommended that SGLT‐2i should be temporarily discontinued in hospitalized patients because of the possibility of euglycemic ketoacidosis and dehydration during COVID‐19 infection.^53^ Two studies included in the pooled data analysis of hospitalization in the SGLT‐2i group vs the non‐SGLT‐2i group revealed an association of decreased incidence of hospitalization in the SGLT‐2i group compared with the non‐SGLT‐2 group.^11^, ^13^ There was no statistically significant effect of SGLT‐2i use as regards ICU admission/mechanical ventilation and mortality. This finding is reinforced by an RCT comparing dapagliflozin to placebo among persons hospitalized with COVID‐19 finding that dapagliflozin was not associated with improvement or adverse events.^14^ In patients with COVID‐19, SGLT‐2i may improve cardiovascular risk factors, including blood pressure, ambient glucose levels, weight, and cardiac function, along with anti‐inflammatory effects, and it is possible that SGLT‐2i might affect viral entry and infection.^52^, ^54^

There are multiple and pleiotropic effects of SGLT‐2i, including an improved endothelial function that may contribute to the reduction of thromboembolic complications as well as anti‐inflammatory effects reducing inflammation markers like interleukin 6 (IL‐6), ferritin, or C‐reactive protein and reducing the intensity of the cytokine storm.^55^

Our analysis showed that GLP‐1RA was associated with a statistically significant reduction in mortality rate, ICU admission/mechanical ventilation, and hospitalization.^11^, ^13^, ^21^, ^24^, ^25^, ^27^, ^28^ Mechanism(s) for these beneficial effects are unclear but may be explained by or more of the following. It has been shown that GLP‐1RA might reduce viral entry and infection in animal models.^52^ The effects of GLP‐1 RA on chronic inflammatory diseases, including nonalcoholic fatty liver disease and atherosclerosis, are mediated by reduced activity of inflammatory pathways, which may affect COVID‐19.^56^ In a study on mice, GLP‐1RA was shown to have an anti‐inflammatory effect by reducing cytokine production and mucus secretion and preserving respiratory function.^57^ GLP‐1RA has antiobesity effects,^58^ which may ameliorate low‐grade chronic inflammation. Finally, it may improve obesity‐associated decreased vitamin D bioavailability and gut microbiome dysbiosis.^59^

GLP‐1RA may also have antithrombotic and mitochondrial protective effects.^60^ GLP‐1 and ACE2 interact in a manner that has been the subject of considerable debate.^60^, ^61^ However, ACE2 upregulation induced by GLP‐1RA may, paradoxically, ameliorate lung injury during COVID‐19 despite enabling virus entry into host target cells.^59^, ^60^, ^61^ Through binding to GLP‐1R, GLP‐1RA inhibits protein kinase C and NF‐kB activation, decreasing the expression of NOD‐like receptors (NLRs) family pyrin domain containing 3 (NLRP3), IL‐1β, tumor necrosis factor‐α, IL‐6, vascular cell adhesion molecule 1, interferon‐γ, and monocyte chemoattractant protein‐1.^59^

A strength of our analysis is the inclusion of different AGMs used during COVID‐19. Second, our pooled analysis compares each group with all other groups. Third, our analysis included data of patients from a wide range of countries. This study has several limitations. The results of the analysis indicated moderate to high heterogeneity. The baseline level of glycosylated hemoglobin before infection with COVID‐19 was not reported in the included studies. Limited information is available regarding other comorbidities, such as chronic obstructive pulmonary disease, obesity, asthma, cardiovascular diseases, and renal function. Most of the included studies involved patients during the early stages of the COVID‐19 pandemic when anticoagulation and steroid treatment were not widely used. The study protocol was not registered in the International Prospective Register of Systematic Reviews (PROSPERO).

COVID‐19 infection can be prevented through infection control measures, vaccination, hand hygiene, mask wear, and maintaining social distancing. Controlling glycemic levels will help reduce the severity of COVID‐19 infection. The analysis suggests that therapy such as GLP1‐RA metformin and SGLT‐2i should be considered in populations with a high prevalence of COVID‐19.

## 5 CONCLUSIONS

Our analysis showed the use of metformin or GLP‐1RA to be associated with decreased risk of mortality. There was an increased risk of ICU admission/mechanical ventilation associated with using DPP‐4i and a decrease associated with using GLP‐1RA. There was an increased risk of hospitalization associated with DPP‐4i and a decrease with SGLT‐2i and GLP‐1RA. Because heterogeneity was moderate to high among the included studies, we cannot recommend discontinuing any group of AGMs. More prospective studies on AGMs with COVID‐19 are needed.

## AUTHOR CONTRIBUTIONS

Conceptualization: Anoop Misra, Zachary Bloomgarden. Screening and data extraction: Mahmoud Nassar, Hazem Abosheaishaa; data check: Awadhesh Kumar Singh; reviewing: Awadhesh Kumar Singh, Anoop Misra, Zachary Bloomgarden; statistical analysis: Mahmoud Nassar; writing the discussion: Mahmoud Nassar. Reviewing: Anoop Misra, Zachary Bloomgarden, and Awadhesh Kumar Singh.

## FUNDING INFORMATION

No funding agent or sponsor for this article.

## CONFLICT OF INTEREST

There is no conflict of interest to declare.

## ETHICAL STATEMENT

No human or animal studies were involved in this study.

## PATIENT AND PUBLIC INVOLVEMENT SUBSECTION

Patients and the public were not involved in any way in this study.

## Supporting information


**DATA S1:** Supporting Information.
