# Supplementary material for: Noninsulin‐based antihyperglycemic medications in patients with diabetes and COVID‐19: A systematic review and meta‐analysis
Source: J Diabetes. 2023 Jan 23;15(2):86–96. doi: 10.1111/1753-0407.13359 (PMC9934962; doi:10.1111/1753-0407.13359)
Supplement: Supplementary file 1 — DATA S1: Supporting Information. [file JDB-15-86-s001.docx]

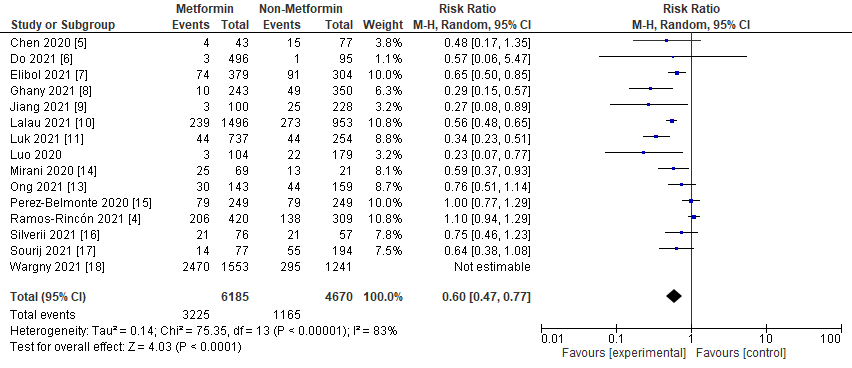


Supplement Figure 1: Forest plot analysis of mortality in patients with diabetes using metformin.


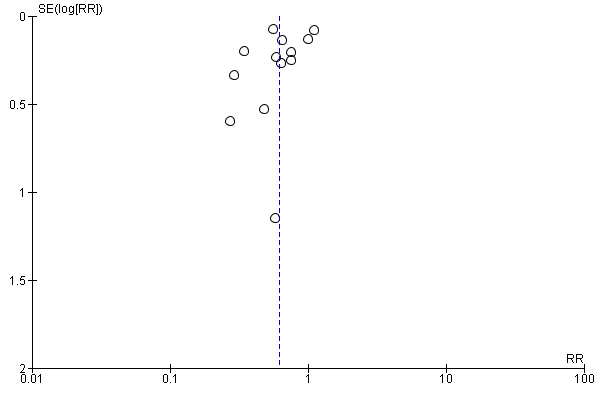


Supplement Figure 2: Funnel plot of mortality in patients with diabetes using Metformin.


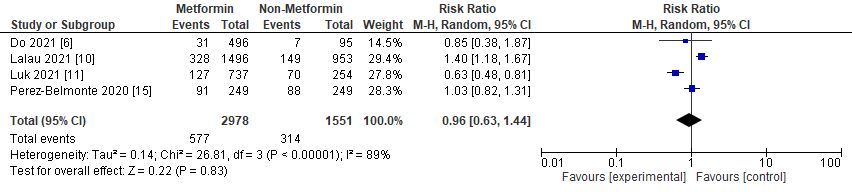


Supplement Figure 3: Forest plot analysis of ICU admission and/or Mechanical ventilation in patients with diabetes using metformin.


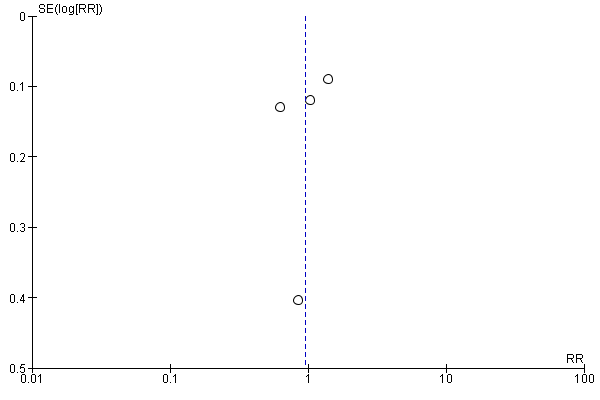


Supplement figure 4: Funnel plot of ICU admission and/or Mechanical ventilation in patients with diabetes using Metformin


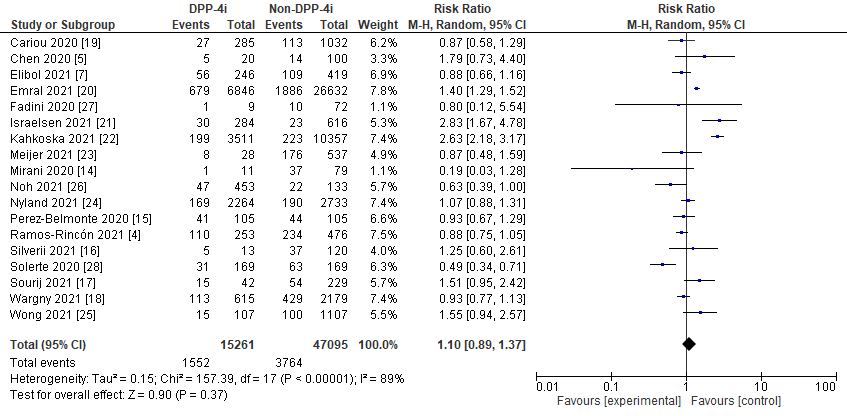


Supplement Figure 5: Forest plot analysis of mortality in patients with diabetes using DPP-4i.


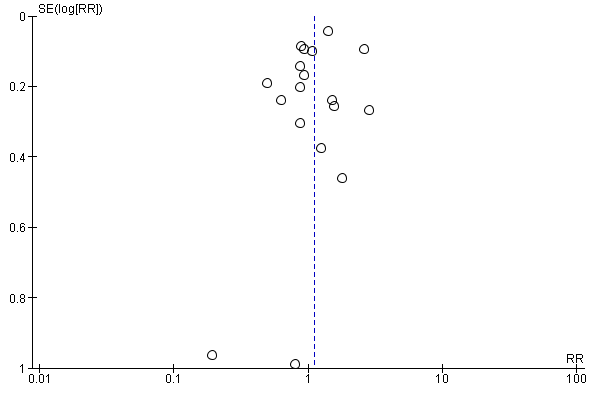


Supplement figure 6: Funnel plot of mortality in patients with diabetes using DPP-4i.


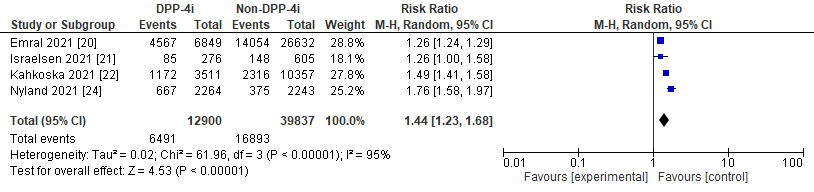


Supplement Figure 7: Forest plot analysis of hospitalization in patients with diabetes using DPP-4i.


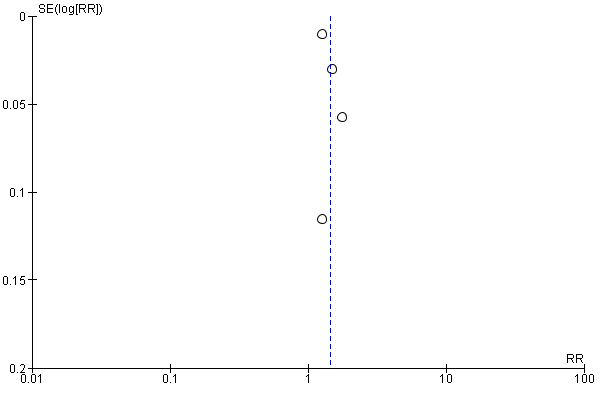


Supplement figure 8: Funnel plot of hospitalization in patients with diabetes using DPP-4i.


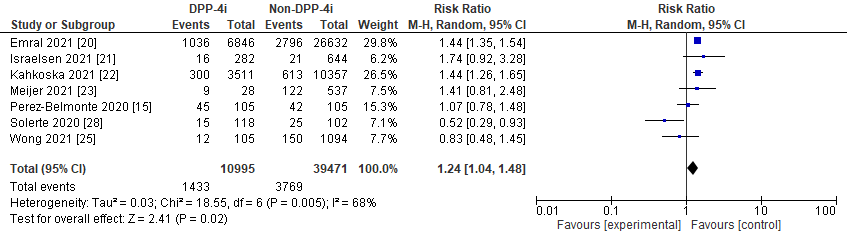


## Supplement Figure 9: Forest plot analysis of ICU admission and/or Mechanical ventilation

in patients with diabetes using DPP-4i.


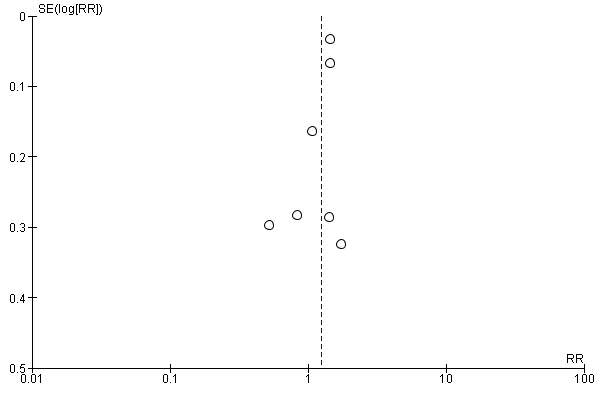


## Supplement figure 10: Funnel plot of analysis of ICU admission and/or Mechanical ventilation

in patients with diabetes using DPP-4i.


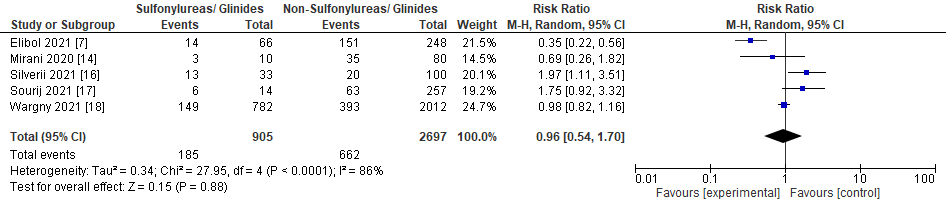


Supplement Figure 11: Forest plot analysis of mortality in patients with diabetes using sulfonylureas/meglitinides.


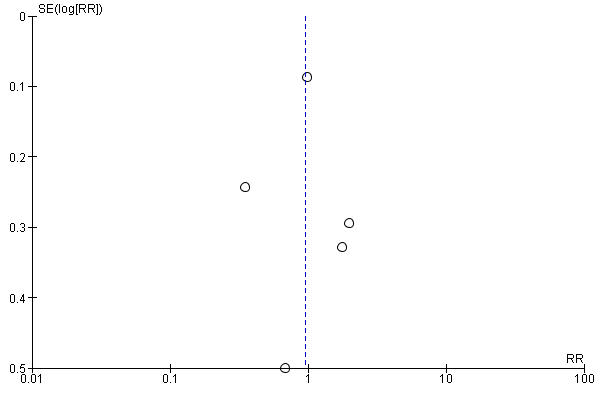


Supplement figure 12: Funnel Plot analysis of mortality in patients with diabetes using sulfonylureas/meglitinides.


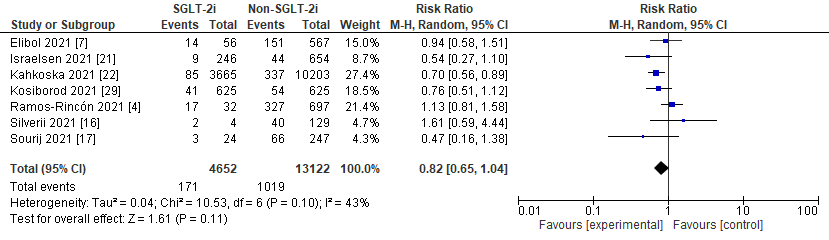


Supplement Figure 13: Forest plot analysis of mortality in patients with diabetes using SGLT-2i


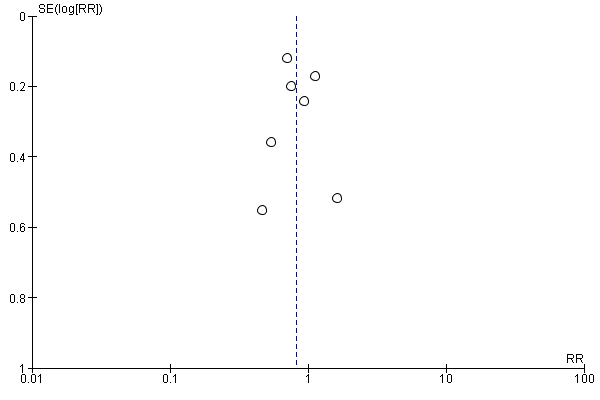


Supplement figure 14: Funnel Plot of mortality in patients with diabetes using SGLT2i


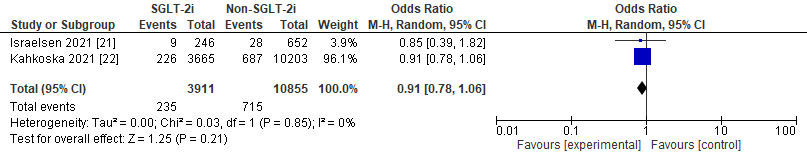


Supplement Figure 15: Forest plot analysis of ICU admission and/or mechanical ventilation in patients with diabetes using SGLT-2i.


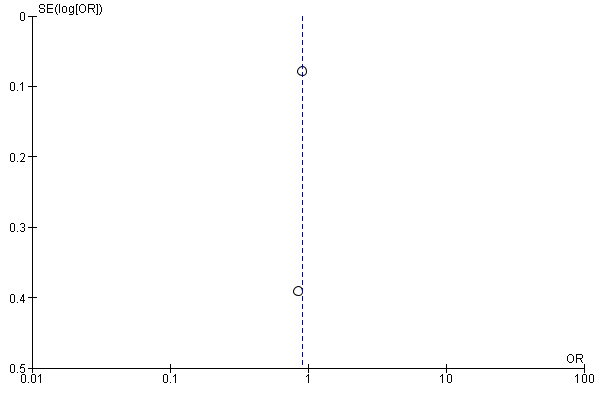


Supplement figure 16: Funnel plot of ICU admission and/or mechanical ventilation in patients with diabetes using SGLT2i.


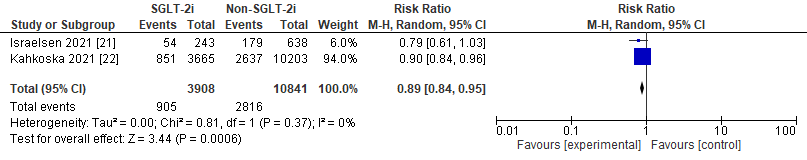


Supplement Figure 17: Forest plot analysis of hospitalization in patients with diabetes using SGLT-2i.


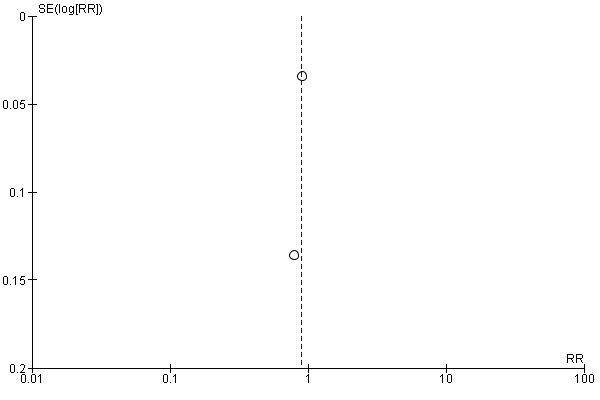


Supplement figure 18: Funnel plot of hospitalization in patients with diabetes using SGLT2i.


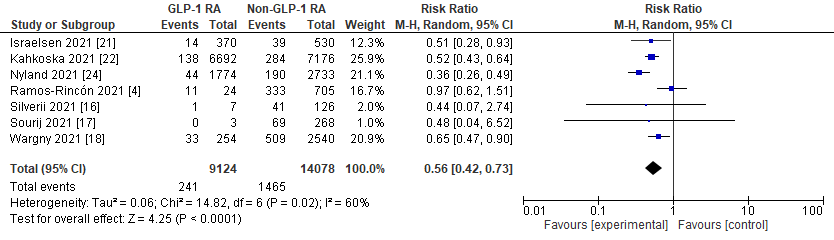


Supplement Figure 19: Forest plot analysis of mortality in patients with diabetes using GLP-1RA.


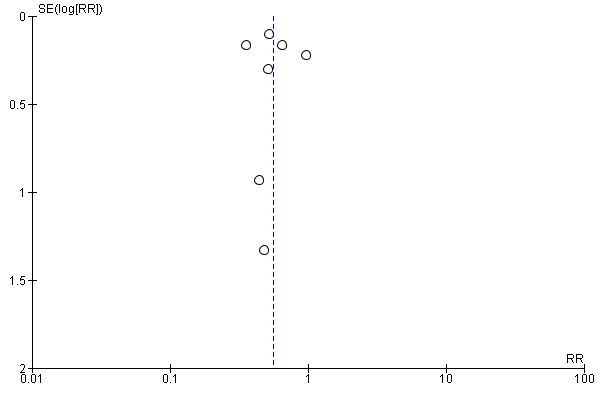


Supplement figure 20: Funnel plot of mortality in patients with diabetes using GLP-1RA.


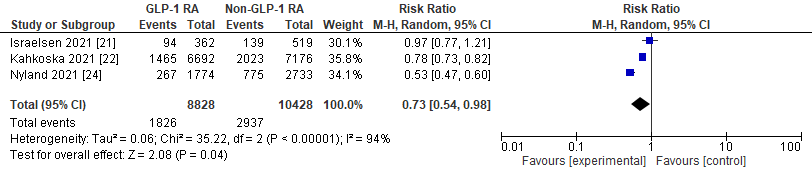


Supplement Figure 21: Forest plot analysis of hospitalizations in patients with diabetes using GLP-1RA.


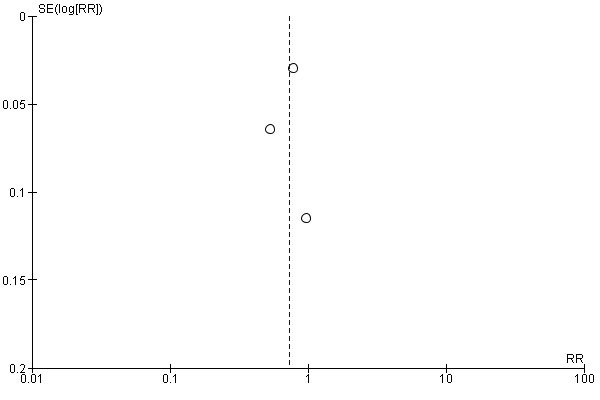


Supplement figure 22: Funnel plot of hospitalizations in patients with diabetes using GLP-1RA.


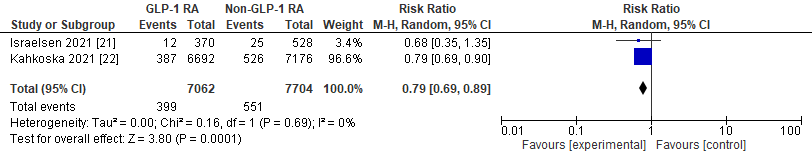


Supplement Figure 23: Forest plot analysis of ICU admission and/or Mechanical ventilation in patients with diabetes using GLP-1RA.


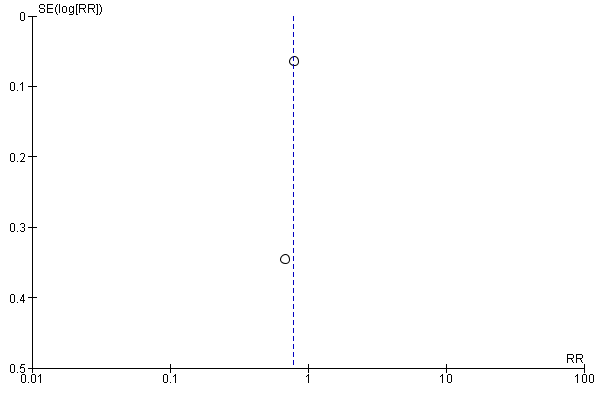


Supplement figure 24: Forest plot analysis of ICU admission and/or Mechanical ventilation in patients with diabetes using GLP-1RA.


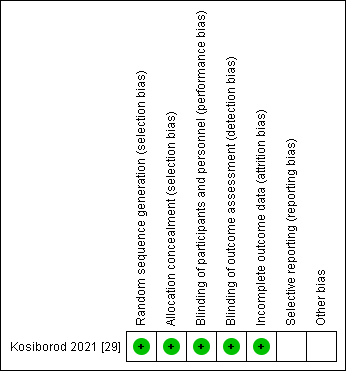


Supplement figure 25: Risk of bias summary about each risk of bias item for of randomized control trial included study.

| **Study Name** | **Selection** | | | | **Comparability** | **Outcome** | | | **Total score** | **Evaluation** |
| --- | --- | --- | --- | --- | --- | --- | --- | --- | --- | --- |
|  | A | B | C | D | E | F | G | H | Maximum 9 |  |
| Cariou et al.,2020 [19] | * | * | * | * | ** | * | * | * | 9 | Good quality |
| Chen et al., 2020 [5] | * | * | * | * | ** | * | * | * | 9 | Good quality |
| Do et al., 2021 [6] | * | * | * | * | ** | * | * | * | 9 | Good quality |
| Elibol et al, 2021 [7] | * | * | * | * | ** | * | * | * | 9 | Good quality |
| Emral et al, 2021 [20] | * | * | * | * | ** | * | * | * | 9 | Good quality |
| Fadini et al., 2020 [27] | * | * | * | * | ** | * | * | * | 9 | Good quality |
| Ghany et al., 2021 [8] | * | * | * | * | ** | * | * | * | 9 | Good quality |
| Israelsen et al, 2021 [21] | * | * | * | * | ** | * | * | * | 9 | Good quality |
| Jiang et al., 2021 [9] | * | * | * | * | ** | * | * | * | 9 | Good quality |
| Kahkoska 2021 et al., [22] | * | * | * | * | ** | * | * | * | 9 | Good quality |
| Lalau et al., 2021 [10] | * | * | * | * | ** | * | * | * | 9 | Good quality |
| Luk 2021 et al., [11] | * | * | * | * | ** | * | * | * | 9 | Good quality |
| Luo 2020 et al., [12] | * | * | * | * | ** | * | * | * | 9 | Good quality |
| Meijer et al, 2021 [23] | * | * | * | * | ** | * | * | * | 9 | Good quality |
| Mirani et al., 2020 [14] | * | * | * | * | ** | * | * | * | 9 | Good quality |
| Noh et al., 2021 [26] | * | * | * | * | ** | * | * | * | 9 | Good quality |
| Nyland et al, 2021 [24] | * | * | * | * | ** | * | * | * | 9 | Good quality |
| Ong et al., 2021 [13] | * | * | * | * | ** | * | * | * | 9 | Good quality |
| Perez-Belmonte et al., 2020 [15] | * | * | * | * | ** | * | * | * | 9 | Good quality |
| Ramos-Rincón et al., 2021 [4] | * | * | * | * | ** | * | * | * | 9 | Good quality |
| Silverii et al, 2020 [16] | * | * | * | * | ** | * | * | * | 9 | Good quality |
| Solerte et al, 2020 [28] | * | * | * | * | ** | * | * | * | 9 | Good quality |
| Sourij et al, 2021 [17] | * | * | * | * | ** | * | * | * | 9 | Good quality |
| Wargny et al., 2021 [18] | * | * | * | * | ** | * | * | * | 9 | Good quality |
| Wong et al., 2021 [25] | * | * | * | * | ** | * | * | * | 9 | Good quality |

A: Representativeness of the exposed cohort, B: Selection of the non-exposed cohort, C: Ascertainment of exposure, D: Demonstration that outcome of interest was not present at start of study, E: Comparability of cohorts on the basis of the design or analysis controlled for confounders, F: Assessment of outcome, G: Was follow-up long enough for outcomes to occur, H: Adequacy of follow-up of cohorts.

**Supplement table 1: Newcastle-Ottawa Quality Assessment Form for retrospective Cohort Studies.**
